# Valsartan Regulates PI3K/AKT Pathways through lncRNA GASL1 to Improve Isoproterenol-Induced Heart Failure

**DOI:** 10.1155/2022/1447399

**Published:** 2022-01-06

**Authors:** Jian Zhou, Xiujuan Duan, Jibing Wang, Yunhong Feng, Jiangyong Yuan

**Affiliations:** ^1^Department of Cardiovascular Medicine, Shanghai East Hospital, Tongji University School of Medicine, Shanghai, China; ^2^The Second Department of Cardiology, The Eighth People's Hospital of Hengshui City, Hengshui, China; ^3^Cardiopulmonary Function Room, People's Hospital of Rugao City, Rugao, China; ^4^The Second Department of Cardiology Affiliated to Hebei University of Engineering, Handan, China

## Abstract

**Objective:**

This study is aimed at determining the expression and function of the GASL1 and PI3K/AKT pathways in isoproterenol- (ISO-) induced heart failure (HF). To determine the moderating effect of valsartan (VAL) on the progression of ISO-induced HF and to elucidate the related mechanism.

**Materials and Methods:**

First, in *in vivo* experiment, we examined the effect of VAL on cardiac function in rats with ISO-induced HF. Similarly, quantitative real-time polymerase chain reaction (qRT-PCR) and Western blot were used to detect the effect of VAL on ISO-treated rat primary cardiomyocytes. Then, si-GASL1-transfected primary cardiomyocytes were constructed and Ad-si-GASL1 was injected through rat tail vein to achieve the effect of lowering GASL1 expression, so as to investigate the role of GASL1 in VAL's treatment of ISO-induced HF.

**Results:**

In ISO-induced HF rat models, the GASL1 decreased while PI3K and p-AKT expressions were abnormally elevated and cardiac function deteriorated, and VAL was able to reverse these changes. In primary cardiomyocytes, ISO induces apoptosis of cardiomyocytes, and expression of GASL1 decreased while PI3K and p-AKT were abnormally elevated, which can be reversed by VAL. The transfection of primary cardiomyocytes with si-GASL1 confirmed that GASL1 affected the expression of PI3K, p-AKT, and the apoptosis of primary cardiomyocytes. Rat myocardium injected with Ad-si-GASL1 was found to aggravate the cardiac function improved by VAL.

**Conclusions:**

This study was the first to confirm that VAL improves ISO-induced HF by regulating the PI3K/AKT pathway through GASL1. And this study demonstrated a significant correlation between HF, VAL, GASL1, and the PI3K/AKT pathway.

## 1. Introduction

Heart failure (HF) is a clinical syndrome of fluid retention, dyspnea, and fatigue caused by impaired ventricular filling and bleeding capacity caused by various functional or structural heart diseases [1]. The cardiovascular disease report released by China provides data that shows that China has 290 million cardiovascular patients and 4.5 million patients with HF [2]. The cardiovascular mortality rate still ranks first, higher than that of tumors and other diseases. The total number of HF patients worldwide is over 26 million, and the incidence of HF continues to rise with the extension of human life [3]. HF has become a chronic disease endangering public health and economic interests. The pathogenesis and effective treatment of HF have also become important contents of scientific research and clinical work. Isoproterenol (ISO) is a major adrenergic receptor agonist. Large doses can damage the heart muscle and cause diastolic and systolic dysfunction, and ISO is widely used in the manufacture of models of HF [4].

The human genome is only about 2% of the total genome, while the remaining 98% can be transcribed into many noncoding or weakly coding RNA molecules of varying lengths, known as noncoding RNAs (ncRNAs) [5]. According to length, ncRNAs are divided into microRNA (miRNA) and long noncoding RNA (lncRNA). ncRNAs larger than 200 nt are called long noncoding RNAs. In the early stage, lncRNA was considered to have no biological function due to poor technical means. With the development of high-throughput technology and the deepening of scientific research, more and more long noncoding RNAs have been discovered, named, and studied. With the deepening of research, the biological functions of lncRNA were gradually disclosed. lncRNA can regulate target genes at various levels, participate in chromosomal dose compensation [6] and genomic imprinting [7], regulate cell cycle [8] and cytoplasmic transmission [9], and regulate cell differentiation and maintenance of stem cells [10]. Many lncRNAs have been proved to have a regulatory effect on various physiological and pathological processes such as the heart and kidney. A new lncRNA was recently discovered, named GASL1 (growth-arrest-associated lncRNA 1). GASL1 can regulate the cell cycle and participate in cell proliferation and colony formation [11, 12]. Other studies have found that the expression of lncRNA GASL1 is downregulated in chronic HF and correlated with cell apoptosis [13]. In this study, GASL1 was selected as one of the research objects to explore its role in isoproterenol cardiac failure.

Phospholipid inositol (PI3K) is a cytoplasmic lipid kinase, and AKT is a key kinase (serine/threonine kinase) in the PI3K pathway. AKT activation requires membrane interaction and phosphorylation of serine 473 (AKT-Ser473) and threonine 308Akt (AKT-Thr308). After multiple stimuli, the pH domain interacts with phosphatidylinositol 3,4,5-triphosphate (PIP3) produced by PI3K to allow cytoplasmic AKT to enter the plasma membrane [14]. Then, AKT-PIP3 interaction, through the interdomain conformational change-induced AKT for open conformer, exposes Thr308 and Ser473 subsequent phosphorylation of phosphoinositide-dependent protein kinase, and phosphorylation of Thr308 and Ser473 fully activated AKT, which through the phosphorylation of the downstream kinases, participate in a variety of regulating cell proliferation and growth, and it also has played an important role in many fields such as cancer [15], angiogenesis [16], and osteoporosis [17].

Valsartan (VAL) was originally an antihypertensive drug but was later used in the treatment of symptomatic HF patients and played a huge role in the development of HF [18]. As early as 2005, Majani confirmed that VAL can also dramatically reduce the decline in the quality of life of patients with HF [19]. In addition, the Maggioni equivalent year study found that adding VAL to prescribed treatments can reduce the incidence of atrial fibrillation [20]. There are many clinical studies on VAL in the treatment of HF, but guidelines often refer to VAL as an intolerable alternative to angiotensin-converting enzyme inhibitors (ACEI). VAL provides an effective means for the treatment of HF. It is widely used in clinical practice and is well known for its cardioprotective effect.

The aim of this study was to determine the function of GASL1 and PI3K-AKT in myocardial tissue and myocardial cells in patients with ISO-induced HF. And explore the effectiveness and related mechanisms of VAL in the treatment of ISO-induced HF.

## 2. Materials and Method

### 2.1. Animal Experiment

24 male SPF 8-week-old healthy male rats (Shanghai East hospital, Tongji University School of Medicine Animal Center, Shanghai, China) weighing 240-260 g were used. They were reared at 25°C and 50% humidity and freely fed. Rats were randomly divided into 3 groups: the sham group, ISO-induced HF model group, and ISO-induced HF+VAL treatment group. We prepared a rat model of HF by giving ISO (Tianpu Biochemical Pharmaceutical, Guangzhou, China) 2 times of doses of 170 mg/kg at 24 h intervals. Rats in the sham group were injected with an equal volume of sterile saline, and rats in the ISO+VAL treatment group were treated with ISO and treated with VAL (Tianpu Biochemical Pharmaceutical, Guangzhou, China) at 30 mg/kg/d for 4 weeks. All rats were housed in cages of appropriate size. Rat myocardial tissues were excised and immediately stored at -80°C for further experiments. This study was approved by the Animal Ethics Committee of Shanghai East hospital, Tongji University School of Medicine Animal Center.

### 2.2. Heart Function Test in Rats

After disinfecting the abdomen of rats, the rats were anesthetized with 0.1% pentobarbital by intraperitoneal injection. The anesthetized rats fixed their limbs and head on the operation platform. Heparin (2 mg/ml, Shanghai East hospital, Tongji University School of Medicine Animal Center, Weihui, China) was injected through the tail vein. After heparinization, we prepared the skin and sterilized the surgical area, took a midline incision of the neck to cut the skin, bluntly separated the layers of tissue, exposed and freed the right common carotid artery, and ligated the distal end of the carotid artery with a surgical line. A rat artery clamp was used to clamp the proximal heart of the carotid artery, and a gap was cut along the common carotid artery at a 30° angle with microscissors under a microscope, and the PV catheter (Nuohai Life Science, Shanghai, China) with an electrode at the front end was inserted into the rat aorta through the cut gap and extended to the left ventricle. Continuously, the pressure-volume data was recorded for 10 min. Hemodynamic data was analyzed using pressure-volume analysis software to obtain the maximal rate of the increase of left ventricular pressure dP/dtmax and the maximal rate of the decrease of left ventricular pressure, dP/dtmax, and the left ventricular systolic pressure (LVSP), and the left ventricular end-diastolic pressure (LVEDP).

### 2.3. Primary Cardiomyocyte Isolation and Cell Culture

The heart was removed using elbow forceps and placed in sterile prechilled D-Hank's solution (Camilo Biological, Nanjing, China). Ophthalmic scissors were used to cut the myocardium into small pieces. The prechilled D-Hank's solution was used to wash the cut myocardial tissue 3-4 times and then transferred to a sterile conical flask containing 10 ml of prechilled trypsin (Camilo Biological, Nanjing, China) and sealed in a 4°C refrigerator overnight. After 12-16 h, we added precooled 10 ml Dulbecco's modified Eagle's medium (DMEM) (Life Technology, Wuhan, China) (complete medium) containing 10% fetal bovine serum (FBS) (Life Technology, Wuhan, China) and penicillin-streptomycin (Life Technology, Wuhan, China) for 10 min and then incubated for 30 min in a 37°C, 5% CO_2_ incubator. We aspirated the supernatant and added 10 ml of type II collagenase (Camilo Biological, Nanjing, China) to digest myocardial tissue and then placed it in a 37°C water bath for 15 min and transferred the supernatant to a sterilized centrifuge tube and added complete medium. 10 ml of type II collagenase was added to the remaining myocardial tissue to redigest the myocardial tissue. The cell suspension obtained by two digestions was centrifuged. Then, we discard the supernatant and resuspend the cell pellet by adding prewarmed complete medium at 37°C. Cell suspensions were seeded into cell culture dishes, and 5 × 10^5^ cells were inoculated in each culture dish. Then, we cultured in an incubator for 1 h and waited for the fibroblasts to adhere to the wall; then, we aspirated the supernatant and washed them again with complete medium to collect the cardiomyocytes attached to the surface of the fibroblasts. The adherence screening process above was repeated once. The screened cells were collected, and the concentration of neonatal rat cardiomyocytes was adjusted to 5 × 10^5^/ml with 37°C prewarmed complete culture medium. BrdU (Camilo Biological, Nanjing, China) was added to the suspension of neonatal rat cardiomyocytes to inhibit the proliferation of fibroblasts. Then, we inoculated it into a culture dish and placed it in an incubator for 48 h. It was washed once with DMEM and then changed the complete medium. The medium was changed once a day and adhered to the culture for 72 h. When the cell fusion appears as a whole beat, we switched to serum-free medium culture. The concentration of 2 *μ*mol/l was used for the construction of ISO-induced HF model. And concentration of 10 *μ*mol/l was used for the construction of VAL.

### 2.4. Cell Transfection

Cells were harvested 48 h after transfection for further analysis. To silence GASL1, a GASL1 siRNA or a control siRNA was transfected by using RNAimax (Invitrogen) following the manufacturer's manuscript. Cells were harvested 72 h after transfection for further analysis.

### 2.5. RNA Isolation and Quantitative Real-Time Polymerase Chain Reaction (qRT-PCR)

The total RNA extraction kit (Thermo Fisher Scientific, Waltham, MA, USA) was used to extract the RNA of the rat's heart tissue and cellular RNA and then reversed transcribed into cDNA for later use. We found the glyceraldehyde 3-phosphate dehydrogenase (GAPDH), lnc-GASL1 sequence on the NCBI website. The cDNA was amplified by a two-step method using an RT-PCR instrument. The reaction system was 20 *μ*l. The amplification conditions were as follows: first denaturation at 95°C for 10 min; then denaturation at 95°C for 15 s, and annealing at 60°C for 1 min, and this step was repeated 40 times. The GADPH housekeeping gene was used as an internal reference control, and the target gene transcription level was calculated by formula 2^-*ΔΔ*Ct^. Primers used were shown in Table 1.

### 2.6. Western Blot

Cut myocardial tissue into small pieces or added lysate (Camilo Biological, Nanjing, China) to cells in a 6-well plate, homogenized until completely lysed, centrifuged at 40°C, 12000 g for 15 min, took the supernatant for protein quantification, and performed polyacrylamide gel electrophoresis (PAGE) and transferred film, 5% skim milk powder blocked for 1 h. Diluted primary antibody (PI3K 1 : 1000 Abcam, AKT 1 : 1000 Abcam, p-AKT 1 : 1000 Abcam, cleaved-caspase-3 1 : 2000 Abcam, GAPDH 1 : 2000 Abcam, Cambridge, MA, USA) incubated with the membrane for 6 h. It was washed with TBST 3 times and diluted the HRP-labeled secondary antibody (Yifei Xue Biotechnology, Nanjing, China) 1: 1000; then, it was incubated with membrane 37°C for 1 h. The TBST was washed 3 times; the electrochemiluminescence (ECL) luminescent solution (Thermo Fisher Scientific, Waltham, MA, USA) was developed, and the scanning analysis was performed with a Tanon-5200 imaging system (Bio, Hercules, CA, USA).

### 2.7. Statistical Analysis

All data are expressed as the mean ± standard deviation (mean ± SD). Differences between two groups were analyzed by using Student's *t*-test. Comparison between multiple groups was done using one-way ANOVA test followed by post hoc test (least significant difference). Statistical analysis was performed using Statistical Product and Service Solutions (SPSS) 22.0 software (IBM, Armonk, NY, USA). When *p* < 0.05, the differences between the groups were statistically significant.

## 3. Results

### 3.1. Effect of VAL on Cardiac Function in Rats with ISO-Induced HF

**Figure 1:**
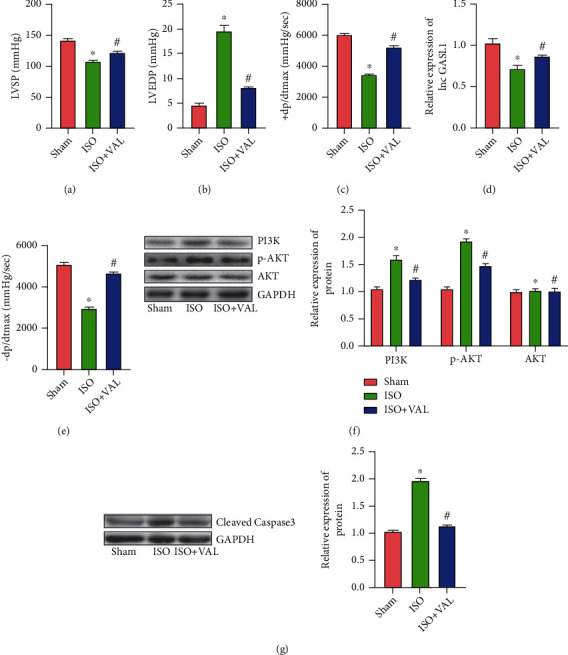
Effect of VAL on cardiac function in rats with ISO-induced HF. (a–d) Results of cardiac function (LVSP, LVEDP, +dP/dtmax, and -dP/dtmax) of rats in different treatment groups (“^∗^” indicates that compared with the sham group, “^#^” indicates that compared with the ISO group *p* < 0.05). (e) The expression levels of lnc GASL1 in rat heart tissues (“^∗^” indicates that compared with the sham group, “^#^” indicates that compared with the ISO group *p* < 0.05). (f) Western blot bands and gray value analysis of PI3K, AKT, and p-AKT (“^∗^” indicates that compared with the sham group, “^#^” indicates that compared with the ISO group *p* < 0.05). (g) Western blot bands and gray value analysis of cleaved-caspase-3 (“^∗^” indicates that compared with the sham group, “^#^” indicates that compared with the ISO group *p* < 0.05).

We divided the rats into three groups, namely, the sham group, ISO group, and ISO+VAL group. Compared with the sham group, the ISO group showed decreased LVSP, increased LVEDP, and decreased ± dP/dtmax. In contrast, when VAL was used to treat ISO-induced HF, the cardiac function indicators above alleviated (Figures 1(a)–1(d)). In addition, the expression of GASL1 in the heart decreased dramatically after ISO treatment and increased after VAL treatment (Figure 1(e)). Meanwhile, we found that the expression of PI3K and p-AKT in the ISO group was dramatically increased, while the expression of PI3K and p-AKT in the heart tissues of the model group was decreased (Figure 1(f)) after VAL treatment, while the expression of AKT was not different among the three groups. At the same time, we examined the apoptosis-related molecules and found that cleaved-caspase-3 expression increased in the heart tissue of rats treated with ISO but decreased in the ISO+VAL group (Figure 1(g)). In ISO-induced HF rats, expression of GASL1 decreased, expression of PI3K, p-AKT, and cleaved-caspase-3 was abnormally elevated, and VAL reversed these changes. VAL has been shown to be effective in the treatment of ISO-induced HF and may be associated with GASL1, PI3K/AKT, and cleaved-caspase-3.

### 3.2. Effect of VAL on ISO-Treated Rat Cardiomyocytes

**Figure 2:**
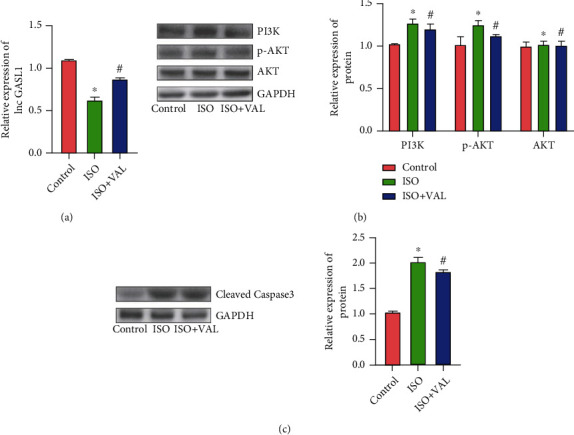
Effect of VAL on ISO-treated rat cardiomyocytes. (a) The expression levels of lnc GASL1 in cardiomyocytes (“^∗^” indicates that compared with the control group, “^#^” indicates that compared with the ISO group *p* < 0.05). (b) Western blot bands and gray value analysis of PI3K, AKT, and p-AKT (“^∗^” indicates that compared with the control group, “^#^” indicates that compared with the ISO group *p* < 0.05). (c) Western blot bands and gray value analysis of cleaved-caspase-3 (“^∗^” indicates that compared with the control group, “^#^” indicates that compared with the ISO group *p* < 0.05).

The results of GASL1 expression detection in primary cardiomyocytes of three groups are shown in Figure 2(a): after ISO treatment of primary cardiomyocytes, GASL1 expression decreased, but the decrease of GASL1 can be inhibited by VAL (Figure 2(a)). At the same time, we examined the expression of PI3K and p-AKT in the three groups of primary cardiomyocytes. The results are shown in Figure 2(b) below: PI3K and p-AKT expression increased after ISO treatment, while VAL inhibited the ISO-induced increase in PI3K and p-AKT expressions in the primary cardiomyocytes, while the expression of AKT showed no difference among the three groups (Figure 2(b)). In addition, cleaved-caspase-3 expression in the three groups was dramatically increased after ISO treatment, while cleaved-caspase-3 expression was dramatically inhibited after intervention with VAL (Figure 2(c)). At the cellular level, it was confirmed that VAL can improve myocardial cell damage caused by ISO. The corresponding changes in the expressions of GASL1, PI3K, and p-AKT were also observed in the experiment, which suggested that GASL1, PI3K/AKT might be involved in the process of the effects of VAL and ISO in cardiomyocytes.

### 3.3. The Effects of GASL1 on VAL in ISO-Treated Primary Rat Cardiomyocytes

**Figure 3:**
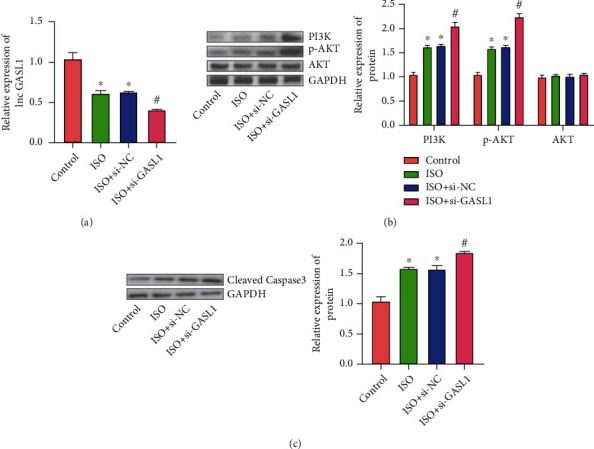
The effects of GASL1 on VAL in ISO-treated primary rat cardiomyocytes. (a) The expression levels of lnc GASL1 in cardiomyocytes (“^∗^” indicates that compared with the control group, “^#^” indicates that compared with the ISO group *p* < 0.05). (b) Western blot bands and gray value analysis of PI3K, AKT, and p-AKT (“^∗^” indicates that compared with the control group, “^#^” indicates that compared with the ISO group *p* < 0.05). (c) Western blot bands and gray value analysis of cleaved-caspase-3 (“^∗^” indicates that compared with the control group, “^#^” indicates that compared with the ISO group *p* < 0.05).

We divided primary cardiomyocytes into 4 groups: control, ISO, ISO+si-NC, and ISO+si-GASL1. The results showed that the expression of GASL1 in ISO-treated rat cardiomyocytes was dramatically decreased. The primary cardiomyocytes of the ISO-treated group were transfected with si-NC, and the expression of GASL1 was not dramatically different from that of the ISO group. However, the expression of GASL1 in rat cardiomyocytes transfected with si-GASL1 dramatically decreased compared with the control group (Figure 3(a)). Transfection of ISO-treated rat cardiomyocytes with si-GASL1 interferes with GASL1 expression and observes the expression of PI3K and P-AKT. The results show that in the ISO-treated rat cardiomyocytes, the PI3K and P-AKT expressions were dramatically increased. And in the ISO+si-NC group, the expression of PI3K and P-AKT had no dramatical difference from that of the ISO group. However, PI3K and P-AKT expression levels in rat cardiomyocytes transfected with si-GASL1 were dramatically higher than that in the ISO group (Figure 3(b)). Similarly, the results showed that cleaved-caspase-3 expression level in ISO-treated rat cardiomyocytes was dramatically increased. And cleaved-caspase-3 expression level in the ISO+si-GASL1 group was dramatically higher than that in the ISO group (Figure 3(c)). It was observed in the experiment that as the expression of GASL1 decreased, the expression of PI3K and P-AKT also changed correspondingly, and accompanied by the change in the degree of cardiomyocyte apoptosis, which confirmed that during the process of ISO-induced cardiomyocyte injury, PI3K/AKT participates as the downstream signaling part of GASL1.

### 3.4. The Effects of VAL and GASL1 on Heart Function in ISO-Induced HF Rats

**Figure 4:**
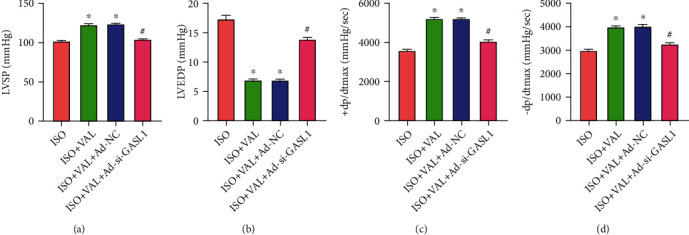
The effects of VAL and GASL1 on heart function in ISO-induced HF rats. (a–d) Results of cardiac function (LVSP, LVEDP, +dP/dtmax, and -dP/dtmax) of rats in different treatment groups (“^∗^” indicates that compared with the ISO group, “^#^” indicates that compared with the ISO+VAL+Ad-NC group *p* < 0.05).

The ISO-induced HF model rats were given VAL or VAL combined with myocardium injection of recombinant adenovirus (Ad-NC or Ad-si-GASL1) to compare the cardiac function results of rats in different treatment groups. Compared with ISO-induced HF model group, rats in the ISO+VAL group had higher level of LVSP, + dP/dtmax, and -dP/dtmax, and with LVEDP decreased. LVSP, +dP/dtmax, and -dP/dtmax were lower than that in the ISO+VAL+Ad-si-GASL1 group compared with ISO+VAL group. However, LVEDP showed the different result (Figures 4(a)–4(d)). We attenuated the myocardial protective effect of VAL by artificially lowering the expression of GASL1 and clinically confirmed the improvement of ISO-induced HF by VAL via GASL1.

## 4. Discussion

VAL is a drug that has been clearly demonstrated to improve myocardial remodeling, and it is not clear whether it is effective in ISO-induced heart HF. Long noncoding RNA is a research hotspot in the cardiovascular field in recent years [21, 22], among which, growth-arrest associated 1 lncRNA (GASL1) has been proved to be related to HF, but other related studies are poor. In this study, animal models and cell (rat primary cardiomyocytes) models of ISO-induced HF were successfully established, and VAL was used for drug intervention to confirm the improvement effect of VAL on ISO-induced HF.

The plasmid si-GASL1 was successfully constructed to transfect the cells, and the upstream and downstream target proteins of the signal transduction pathway were determined, which confirmed that the signal transduction pathways of VAL to improve ISO induced HF were GASL1 and PI3K/AKT. Ad-si-GASL1, an adenovirus vector of GASL1, was successfully constructed and injected into the myocardium of ISO-induced rats to verify the effect of GASL1 on the myocardial protection of VAL. This study is the first to confirm that VAL modulates the PI3K/AKT signaling pathway through GASL1 in ISO-induced HF. GASL1 is a new entry point for the treatment of ISO-induced HF.

Although the significant correlation between HF, VAL, GASL1, and the PI3K/AKT pathway had been confirmed in the experiment, the specific transcription genes and cytokines involved in the signaling process still need to be further clarified. VAL has been shown to interfere with ISO-induced HF; however, other possible mechanisms for VAL in the treatment of HF remain to be developed. GASL1 has been shown to have a protective role in the process of ISO-induced HF, which provides a new entry point for treatment, but specific effective intervention methods need to be developed.

## 5. Conclusion

This study was the first time to confirm that VAL improves ISO-induced HF by regulating the PI3K/AKT pathway through GASL1. And this study demonstrated a significant correlation between HF, VAL, GASL1, and the PI3K/AKT pathway.

## Figures and Tables

**Table 1:** Real time PCR primers.

Gene name	Forward (5′>3′)	Reverse (5′>3′)
lnc GASL1	CTGAGGCCAAAGTTTCCAAC	CAGCCTGACTTTCCCT CTTCT
GAPDH	ACAACTTTGGTATCGTGGAAGG	GCCATCACGCCACAGTTTC

qRT-PCR: quantitative real-time polymerase chain reaction.

## Data Availability

The datasets used and analyzed during the current study are available from the corresponding author on reasonable request.
